# Which Color Channel Is Better for Diagnosing Retinal Diseases Automatically in Color Fundus Photographs?

**DOI:** 10.3390/life12070973

**Published:** 2022-06-28

**Authors:** Sangeeta Biswas, Md. Iqbal Aziz Khan, Md. Tanvir Hossain, Angkan Biswas, Takayoshi Nakai, Johan Rohdin

**Affiliations:** 1 Faculty of Engineering, University of Rajshahi, Rajshahi 6205, Bangladesh; iqbal_aziz_khan@ru.ac.bd (M.I.A.K.); mth_cse@ru.ac.bd (M.T.H.); 2 CAPM Company Limited, Bonani, Dhaka 1213, Bangladesh; angkan@capmbd.com; 3 Faculty of Engineering, Shizuoka University, Hamamatsu 432-8561, Japan; nakai.takayoshi@shizuoka.ac.jp; 4 Faculty of Information Technology, Brno University of Technology, 61200 Brno, Czech Republic; rohdin@fit.vutbr.cz

**Keywords:** color fundus photographs, detection of retinal diseases, deep neural network, segmentation of retinal landmarks

## Abstract

Color fundus photographs are the most common type of image used for automatic diagnosis of retinal diseases and abnormalities. As all color photographs, these images contain information about three primary colors, i.e., red, green, and blue, in three separate color channels. This work aims to understand the impact of each channel in the automatic diagnosis of retinal diseases and abnormalities. To this end, the existing works are surveyed extensively to explore which color channel is used most commonly for automatically detecting four leading causes of blindness and one retinal abnormality along with segmenting three retinal landmarks. From this survey, it is clear that all channels together are typically used for neural network-based systems, whereas for non-neural network-based systems, the green channel is most commonly used. However, from the previous works, no conclusion can be drawn regarding the importance of the different channels. Therefore, systematic experiments are conducted to analyse this. A well-known U-shaped deep neural network (U-Net) is used to investigate which color channel is best for segmenting one retinal abnormality and three retinal landmarks.

## 1. Introduction

Diagnosing retinal diseases at their earliest stage can save a patient’s vision since, at an early stage, the diseases are more likely to be treatable. However, ensuring regular retina checkups for each citizen by ophthalmologists is infeasible not only in developing countries with huge populations but also in developed countries with small populations. The main reason is that the number of ophthalmologists compared to citizens is very small. It is particularly true for low-income and low-middle-income countries with huge populations, such as Bangladesh and India. For example, according to a survey conducted by the International Council of Ophthalmology (ICO) in 2010 [1], there were only four ophthalmologists per million people in Bangladesh. For India, the number was 11. Even for high-income countries with a small population, such as Switzerland and Norway, the numbers of ophthalmologists per million were not very high (91 and 68, respectively). More than a decade later, in 2021, these numbers remain roughly the same. Moreover, 60+ people (who are generally at high risk of retinal diseases) are increasing in most countries. The shortage of ophthalmologists and the necessity of regular retina checkups at low cost inspired researchers to develop computer-aided systems to detect retinal diseases automatically.

Different kinds of imaging technologies (e.g., color fundus photography, monochromatic retinal photography, wide-field imaging, autofluorescence imaging, indocyanine green angiography, scanning laser ophthalmoscopy, Heidelberg retinal tomography and optical coherence tomography) have been developed for the clinical care and management of patients with retinal diseases [2]. Among them, color fundus photography is available and affordable in most parts of the world. A color fundus photograph can be captured using a non-mydriatic fundus camera, handled by non-professional personnel, and delivered online to major ophthalmic institutions for follow-up in the case a disease is suspected. Moreover, there are many publicly available data sets of color fundus photographs such as CHASE_DB1 [3,4], DRIVE [5], HRF [6], IDRiD [7], Kaggle EyePACS data set [8], Messidor [9], STARE [10,11] and UoA_DR [12] to help researchers compare the performances of their proposed approaches. Therefore, color fundus photography is used more widely than other retinal imaging techniques for automatically diagnosing retinal diseases.

In color fundus photographs, the intensity of colors reflected from the retina are recorded in three color channels, red, green, and blue. In this paper, we investigate which color channel is better for the automatic detection of retinal diseases as well as the segmentation of retinal landmarks. Although the detection of retinal diseases is the main objective of computer-aided diagnostic (CAD) systems, segmentation is also an important part of many CAD systems. For example, structural changes in the central retinal blood vessels (CRBVs) may indicate diabetic retinopathy (DR). Therefore, a technique for segmenting CRBVs is often an important step in DR detection systems. Similarly, optic disc (OD) segmentation is important for some glaucoma detection algorithms.

In this work, we first extensively survey the usage of the different color channels in previous works. Specifically, we investigate works on four retinal diseases (i.e., glaucoma, age-related macular degeneration (AMD), and DR, diabetic macular edema (DME)) which are the major causes of blindness [13,14,15,16] as well as works on the segmentation of retinal landmarks, such as OD, macula/fovea and CRBVs, and retinal atrophy. We notice that the focus of the previous works was not to investigate which of the different channels (or combination of channels) is the best for the automatic analysis of fundus photographs. At the same time, there does not seem to be complete consensus on this since different studies used different channels (or combinations of channels). Therefore, to better understand the importance of the different color channels, we develop color channel-specific U-shaped deep neural networks (i.e., U-Nets [17]) for segmenting OD, macula, and CRBVs. We also develop U-Nets for segmenting retinal atrophy. The U-Net is well-known for its excellent performance in medical image segmentation tasks. The U-Net can segment images in great detail, even using very few images in the training phase. It is shown in [17] that a U-Net trained using only 30 images outperformed a sliding window convolutional neural network for the ISBI neuronal structures in the EM stacks challenge 2012.

To the best of our knowledge, a systematic exploration of the importance of different color channels for the automatic processing of color fundus photographs has not been undertaken before. Naturally, a better understanding of the effectiveness of different color channels can reduce the amount of development time of future algorithms. In the long term, it may also affect the design of new fundus cameras and the procedures for capturing fundus photographs, e.g., the appropriate light conditions.

The organization of this paper is as follows: in Section 2, we describe briefly the different color channels of a color fundus photograph, in Section 3, we survey which color channels were used in previous works for the automatic detection of retinal diseases and segmentation. In Section 4, we describe our setup for U-Nets based experiments. In Section 5, we show the performance of color channel-specific U-Nets. At last, in Section 6, we draw conclusions about our findings. Some steps of image pre-processing in more detail and additional experiments are described in the Appendices.

## 2. Fundus Photography

**Figure 1:**
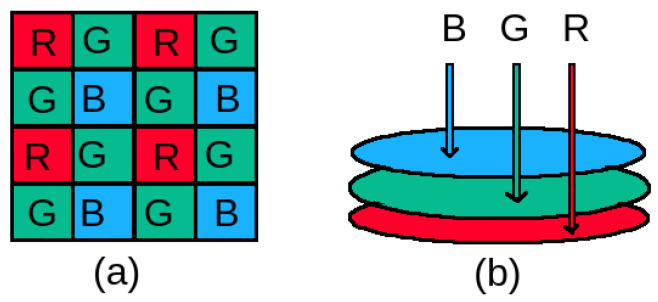
Sensors used in fundus cameras: (**a**) commonly used a single layered sensor coated with a color filter array having a Bayer pattern and (**b**) less commonly used three-layered direct imaging sensor. R: Red, G: Green, B: Blue.

Our retina does not have any illumination power. Moreover, it is a minimally reflective surface. Therefore, a fundus camera which is a complex optical system, needs to illuminate and capture the low reflected light of the retina simultaneously while imaging [18]. A single image sensor coated with a color filter array (CFA) is used more commonly to capture the reflected light in a fundus camera. In a CFA, in general, color filters are arranged following the Bayer pattern [19], developed by the Eastman Kodak company, as shown in Figure 1a. Instead of using three filters for capturing three primary colors (i.e., *red*, *green* and *blue*) reflected from the retina, only one filter is used per pixel to capture one primary color in the Bayer pattern. In this pattern, the number of *green* filters is twice the number of *blue* and *red* filters. Different kinds of demosaicing techniques are applied to get full color fundus photographs [20,21,22]. Some sophisticated and expensive fundus cameras do not use a CFA with a Bayer pattern to distinguish color, rather they use a *direct imaging sensor* with three layers of photosensitive elements as shown in Figure 1b. No demosaicing technique is necessary for getting full color fundus photographs from such fundus cameras.

**Figure 2:**
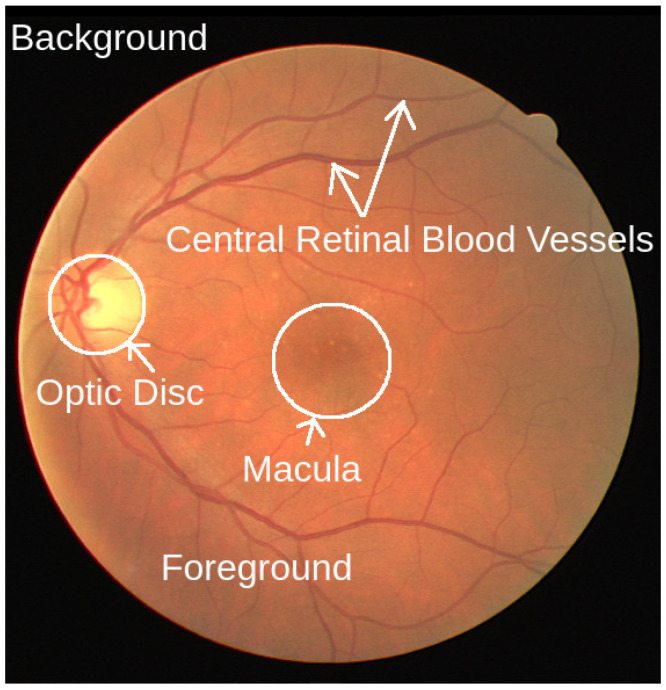
A color fundus photograph. We can see the retinal landmarks, i.e., optic disc, macula, and central retinal blood vessels, on the circular and colored foreground, surrounded by a dark background. Source of image: publicly available DRIVE data set and image file: 21_training.tif.

**Figure 3:**
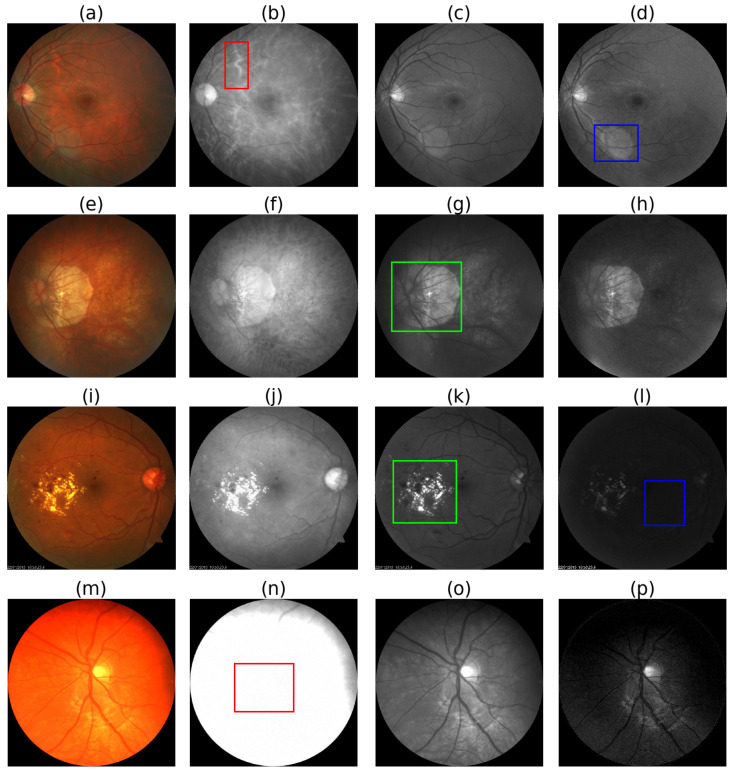
Pros and cons of different color channels. 1st column i.e., (**a**,**e**,**i**,**m**): RGB fundus photographs, 2nd column i.e., (**b**,**f**,**j**,**n**): red channel images, 3rd column i.e., (**c**,**g**,**k**,**o**): green channel images, and 4th column i.e., (**d**,**h**,**l**,**p**): blue channel images. Choroidal blood vessels are clearly visible in the red channel, as shown inside the red box in (**b**). Lens flares are more visible in the blue channel, as shown inside the blue box in (**d**). Atrophy and diabetic retinopathy affected areas are more clearly visible in the green channel as shown inside the green boxes in (**g**,**k**). As shown inside the blue box in (**l**), the blue channel is prone to underexposure. The red channel is prone to overexposure, as shown inside the red box in (**m**). Source of fundus photographs: (**a**) PALM/PALM-Training400/H0025.jpg, (**e**) PALM/PALM-Training400/P0010.jpg, (**i**) UoA_DR/94/94.jpg, and (**m**) CHASE_DB1/images/Image_11L.jpg.

As shown in Figure 2, in a color fundus photograph, we can see the major retinal landmarks, such as the optic disc (OD), macula, and central retinal blood vessels (CRBVs), on the colored foreground surrounded by the dark background. As can be seen in Figure 3, different color channels highlight different things in color fundus photographs. We can see the boundary of the OD more clearly and the choroid in more detail in the red channel. The red channel helps us segment the OD more accurately and see the choroidal blood vessels and choroidal lesions such as nevi or tumors more clearly than the other two color channels. The CRBVs and hemorrhages can be seen in the green channel with excellent contrast. The blue channel allows us to see the retinal nerve fiber layer (RNFL) defects and epiretinal membranes more clearly than the other two color channels.

## 3. Previous Works on Diagnosing Retinal Disease Automatically

Many diseases can be the cause of retinal damage, such as glaucoma, age-related macular degeneration (AMD), diabetic retinopathy (DR), diabetic macular edema (DME), retinal artery occlusion, retinal vein occlusion, hypertensive retinopathy, macular hole, epiretinal membrane, retinal hemorrhage, lattice degeneration, retinal tear, retinal detachment, intraocular tumors, penetrating ocular trauma, pediatric and neonatal retinal disorders, cytomegalovirus retinal infection, uveitis, infectious retinitis, central serous retinopathy, retinoblastoma, endophthalmitis, and retinitis pigmentosa. Among them, glaucoma, AMD, DR, and DME drew the main focus of researchers for color fundus photograph-based automation. One reason could be that for many cases, these causes lead to irreversible complete vision loss, i.e., blindness if they are left undiagnosed and untreated. According to the information reported in [23,24], glaucoma, AMD, and DR are among the five most common causes of vision impairment in adults. Among 7.79 billion people living in 2020, 295.09 million people experienced moderate or severe vision impairment (MSVI) and 43.28 million people were blind. Glaucoma was the cause of MSVI for 4.14 million people, whereas AMD for 6.23 million and DR for 3.28 million people. Glaucoma was the cause of blindness for 3.61 million people, whereas AMD for 1.85 million and DR for 1.07 million people [24]. Therefore, in our literature survey, we investigate the color channels used in previously published studies for automatically diagnosing glaucoma, DR, AMD, and DME. We also survey works on segmentation of retinal landmarks, such as OD, macula/fovea and CRBVs, and retinal atrophy.

We consider both original studies and reviews as the source of information. However, our survey includes only original studies written in English and published in SJR ranked Q1 and Q2 journals. Note that SJR (SCImago Journal Rank) is an indicator developed by SCImago from the widely known algorithm Google PageRank [25]. This indicator shows the visibility of the journals contained in the Scopus database from 1996. We used different keywords such as ‘automatic retinal disease detection’, ‘automatic diabetic retinopathy detection’, ‘automatic glaucoma detection’, ‘detect retinal disease by deep learning’, ‘segment macula’, ‘segment optic disc’, and ‘segment central retinal blood vessels’ in the Google search engine to find previous studies. After finding a paper, we checked the SJR rank of the journal. We used the reference list of papers published in Q1/Q2 journals; we especially benefited from the review papers related to our area of interest.

In this paper, we include our findings based on information reported in 199 journal papers. As shown in Table 1, the green channel dominates non-neural network-based previous works, whereas RGB images (i.e., red, green, and blue channels together) dominate neural network-based previous works. Few works were based on the red and blue channels and they were mainly for atrophy segmentation. See Table 2, Table 3, Table 4, Table 5 and Table 6 for the color channel distribution in our studied previous works.

## 4. Experimental Setup

### 4.1. Hardware & Software Tools

We performed all experiments using TensorFlow’s Keras API 2.0.0, OpenCV 4.2.0, and Python 3.6.9. We used a standard PC with 32 GB memory, Intel 10th Gen Core i5-10400 Processor with six cores per socket, and Intel UHD Graphics 630 (CML GT2).

### 4.2. Data Sets

We used RGB color fundus photographs from seven publicly available data sets: (1) Child Heart Health Study in England (CHASE) data set [3,4], (2) Digital Retinal Images for Vessel Extraction (DRIVE) data set [5], (3) High-Resolution Fundus (HRF) data set [6], (4) Indian Diabetic Retinopathy Image Dataset (IDRiD) [7], (5) Pathologic Myopica Challenge (PALM) data set [218], (6) STructured Analysis of the Retina (STARE) data set [10,11], and (7) University of Auckland Diabetic Retinopathy (UoA-DR) data set [12]. Images in these data sets were captured by different fundus cameras for different kinds of research objectives, as shown in Table 7.

Since all of the seven data sets do not have manually segmented images for all retinal landmarks and atrophy, we cannot use all of them for all kinds of segmentation tasks. Therefore, instead of seven data sets we used five data sets for the experiments of segmenting CRBVs, three data sets for OD, and two data sets for macula, while only one data set for the experiments of segmenting retinal atrophy. We emphasize to have reliable results. For that we used the majority of the data (i.e., 55% of the data) as the test data. We prepared one training and one validation set. By combining 25% of the data from each data set, we prepared the training set, whereas we prepared the validation set by combining 20% of the data from each data set. By taking the rest of the 55% of the data from each data set, we prepared individual test sets for each type of segmentation. See Table 8 for the number of images in the training, validation, and test sets. Note that the training set is used to tune the parameters of the U-Net (i.e., weights and biases), the validation set is used to tune the hyperparameters (such number of epochs, learning rate, and activation function), and the test set is used to evaluate the performance of the U-Net.

### 4.3. Image Pre-Processing

We prepared four types of 2D fundus photographs: $I_{R}$, $I_{G}$, $I_{B}$, and $I_{Gr}$. By splitting 3D color fundus photographs into three color channels (i.e., *red*, *green* and *blue*), we prepared $I_{R}$, $I_{G}$, $I_{B}$. Moreover, by performing a weighted summation of $I_{R}$, $I_{G}$, $I_{B}$, we prepared the grayscale image, $I_{Gr}$. By a grayscale image, we generally mean an image whose pixels have only one value representing the amount of light. It can be visualized as different shades of gray. An 8-bit grayscale image has pixel values in the range 0–255. There are many ways to convert a color image into a grayscale image. In this paper, we use a function from the OpenCV library where each grey pixel is generated according to the following scheme: $I_{Gr}=0.299\times I_{R}+0.587\times I_{G}+0.114\times I_{B}$. This conversion scheme is frequently used in computer vision and implemented in different toolboxes, e.g., GIMP and MATLAB [219] including OpenCV.

**Figure 4:**
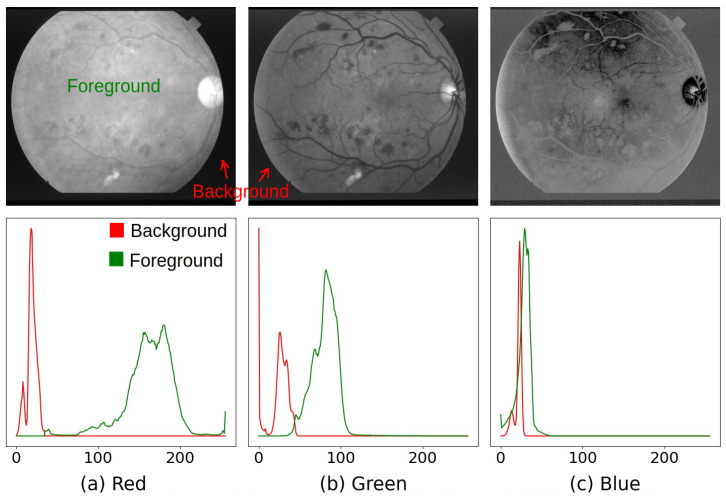
There is a noticeable overlap in the histograms of the foreground and the background in the blue channel. Histograms are slightly overlapped in the green channel. In the red channel, histograms are not overlapped and easily separable. Therefore, by setting 0 to the pixels lower than the threshold value, θ and setting 255 to the pixels higher than the θ, we can easily generate the background mask from the red channeled image. Source of fundus photograph: STARE data set and image file: im0139.ppm.

The background of a fundus photograph does not contain any information about the retina, which can be helpful for manual or automatic retina-related tasks. Sometimes background noise can be misleading. In order to avoid the interference of the background noise in any decision, we need to use a binary background mask, which has zero for the pixels of the background and $2^{n}-1$ for the pixels of the foreground, where *n* is the number of bits used for the intensity of each pixel. For an 8-bit image, $2^{n}-1=255$. Except the DRIVE and HRF data sets, background masks are not provided for the other five data sets. Therefore, we followed the steps described in Appendix A to generate the background masks for all data sets. We generated binary background masks for DRIVE and HRF data sets in order to keep the same set up for all data sets. Overall, $I_{R}$ has a higher intensity than $I_{G}$ and $I_{B}$ in all data sets, whereas $I_{B}$ has a lower intensity compared to $I_{R}$ and $I_{G}$. Moreover, in $I_{R}$, the foreground is less likely to overlap with the background noise than $I_{G}$ and $I_{B}$. In $I_{B}$, the foreground intensity has the highest possibility to be overlapped with the intensity of the background noise, as shown in Figure 4. Therefore, we use $I_{R}$ (i.e., the *red* channel image) for generating the binary background masks.

We used the generated background mask and followed the steps described in Appendix B for cropping out the background as much as possible and removing background noise outside the field-of-view (FOV). Since cropped fundus photographs of different data sets have different resolutions as shown in Table 7, we re-sized all masked and cropped fundus photographs to 256×256 by bicubic interpolation so that we could use one U-Net. After resizing fundus photographs, we applied contrast limited adaptive histogram equalization (CLAHE) [220] to improve the contrast of each single colored image. Then we re-scaled pixel values to [0,1]. Note that, re-scaling pixel values to [0,1] is not necessary for fundus photographs. However, we did it to keep the input and output in the same range. We did not apply any other pre-processing techniques to the images.

Similar to the fundus photographs, reference masks provided by the data sets for segmenting OD, CRBVs and retinal atrophy can have an unnecessary and noisy background. We, therefore, cropped out the unnecessary background of the provided reference masks and removed noise outside the field-of-view area by following the steps described in Appendix B. Since some provided masks are not binary masks, we turned them into 2D binary masks by following the steps described in Appendix C. No data set provides binary masks for segmenting the macula. Instead the center of the macula are provided by the PALM and UoA-DR. We generated binary masks for segmenting macula using the center values of the macula and the OD masks of the PALM and UoA-DR by following the steps described in Appendix D. We re-sized all kinds of binary masks to 256×256 by bicubic interpolation. We then re-scaled pixel values to [0,1], since we used the *sigmoid* function as the activation function in the output layer of the U-Net and the range of this function is [0,1].

### 4.4. Setup for U-Net

We trained color-specific U-Nets with an architecture as shown in Table A3 of Appendix E. To train our U-Nets, we set Jaccard co-efficient loss (JCL) as the loss function; RMSProp with a learning rate of 0.0001 as the optimizer and mini_batch_size=8. We reduced the learning rate if there was no change in the validation_loss for more than 30 consecutive epochs. We stopped the training if the validation_loss did not change in 100 consecutive epochs. We trained all color-specific U-Nets five times to avoid the effect of randomness caused by different factors, including weight initialization and dropout, on the U-Net’s performance. That means, in total, we trained 100 U-Nets, among which 25 U-Nets for OD segmentation (i.e., five models for each RGB, *gray*, *red*, *green*, and *blue*), 25 U-Nets for macula segmentation, 25 U-Nets for CRBVs segmentation, and 25 U-Nets for atrophy segmentation. We estimate the performance of each model separately and then report mean±standard deviation of the performance for each category.

### 4.5. Evaluation Metrics

In segmentation, the U-Net shall predict whether a pixel is part of the object in question (e.g., OD) or not. Ideally, it should therefore output:

$$pixel\_label=\left\{\begin{array}{cc} 1, & \mathrm{If}\;\mathrm{the}\;\mathrm{pixel}\;\mathrm{belongs}\;\mathrm{to}\;\mathrm{the}\;\mathrm{targeted}\mathrm{retinal}\;\mathrm{landmark}\;\mathrm{or}\;\mathrm{atrophy}.\\ 0, & \mathrm{Otherwise}. \end{array}\right.$$

However, instead of 0/1, the output of the U-Net is in the range [0, 1] for each pixel since we use sigmoid as the activation function in the last layer. The output can be interpreted as the probability that the pixel is part of the mask. To obtain a hard prediction (0/1), we use a threshold of 0.5. By comparing the hard prediction to the reference, it is decided whether the prediction is a true positive (TP), true negative (TN), false-positive (FP), or false negative (FN). Using those results for each pixel in the test set, we estimated the performance of the U-Net using four metrics. We used three metrics that are commonly used in classification tasks (i.e., precision, recall, and area-under-curve (AUC)) and one metric which is commonly used in image segmentation tasks (i.e., mean intersection-over-union (MIoU), also known as Jaccard index or Jaccard similarity coefficient). We computed precision = TP / (TP + FP) and recall = TP / (TP + FN) for both semantic classes together. On the other hand, we computed IoU = TP / (TP + FP + FN) for each semantic class (i.e., 0/1) and then averaged over the classes to estimate MIoU. We estimated the AUC for the receiver operating characteristic (ROC) curve using a linearly spaced set of thresholds. Note that AUC is a threshold-independent metric, unlike precision, recall, and MIoU, which are threshold-dependent metrics.

## 5. Performance of Color Channel Specific U-Net

Comparing the results as shown in Table 9, Table 10, Table 11 and Table 12, we can say that the U-Net is more successful at segmenting the OD and less successful at segmenting CRBVs for all channels. The U-Net performs better when all three color channels (i.e., RGB images) are used together than when the color channels are used individually. For segmenting the OD, the *red* and *gray* channels are better than the *green* and *blue* channels (see Table 9). For segmenting CRBVs the *green* channel performs better than other single channels, whereas both the *red* and *blue* channels perform poorly (see Table 10). For macula segmentation, there is no clear winner among *gray* and *green* channels. Although, the *blue* channel is a bad choice for segmenting the CRBVs, it is reasonably good at segmenting macula (see Table 11). For segmenting retinal atrophy, the *green* channel is better than other single channel and the *blue* channel is also a good choice (see Table 12).

**Figure 5:**
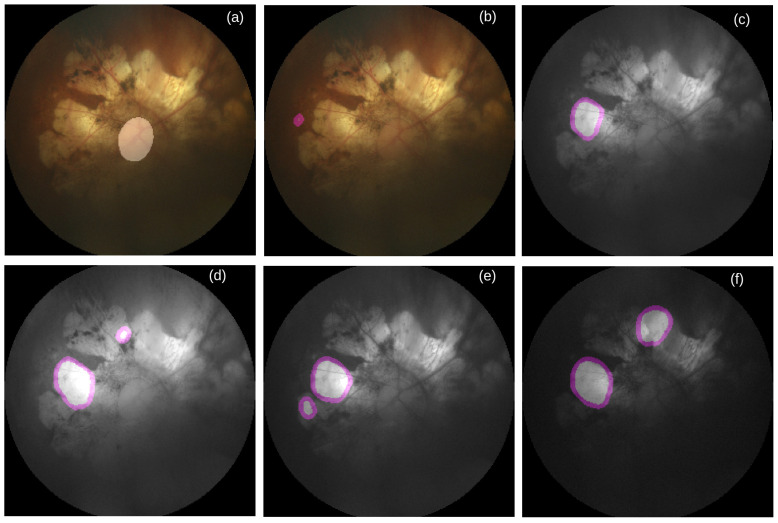
Failure case of OD segmentation. (**a**) RGB image overlaid by reference mask for OD segmentation, (**b**) RGB image overlaid by inaccurately predicted OD mask, (**c**) Grayscale image overlaid by inaccurately predicted mask for OD segmentation, (**d**) Red channel image overlaid by inaccurately predicted mask for OD segmentation, (**e**) Green channel image overlaid by inaccurately predicted mask for OD segmentation, and (**f**) Blue channel image overlaid by inaccurately predicted mask for OD segmentation. Source of image: PALM/P0159.jpg.

**Figure 6:**
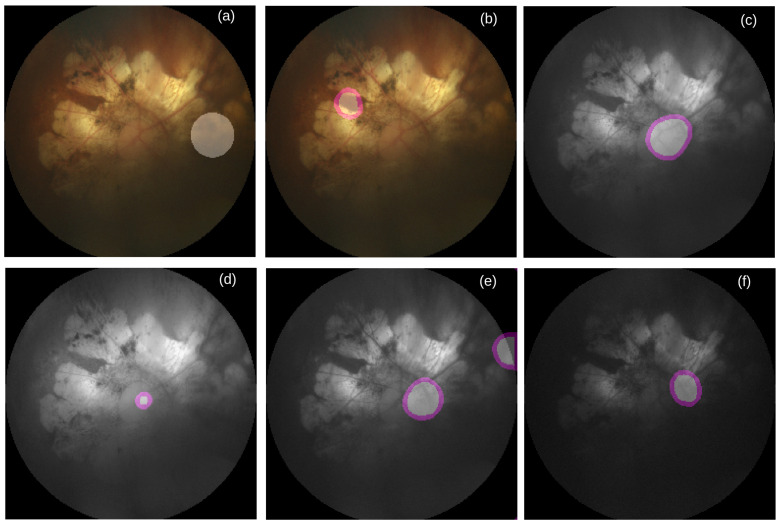
Failure case of macula segmentation. (**a**) RGB image overlaid by reference mask for macula segmentation, (**b**) RGB image overlaid by inaccurate predicted macula mask, (**c**) Grayscale image overlaid by inaccurately predicted mask for macula segmentation, (**d**) Red channel image overlaid by inaccurately predicted mask for macula segmentation, (**e**) Green channel image overlaid by inaccurately predicted mask for macula segmentation, and (**f**) Blue channel image overlaid by inaccurately predicted mask for macula segmentation. Source of image: PALM/P0159.jpg.

To better understand the performance of U-Nets, we manually inspect all images together with their reference and predicted masks. As shown in Table 13, we see that for the majority number of cases, all color-specific U-Nets can generate at least partially accurate masks for segmenting OD and macula. When the retinal atrophy severely affects any retina, no channel-specific U-Net can generate accurate masks for segmenting OD and macula, as shown in Figure 5 and Figure 6. For many cases multiple areas in the generated masks are pointed as OD (see Figure 5d–f) and macula (see Figure 6d). As shown in Table 14, it happens more in the *gray* channel for the macula and in the *green* channel for the OD.

**Figure 7:**
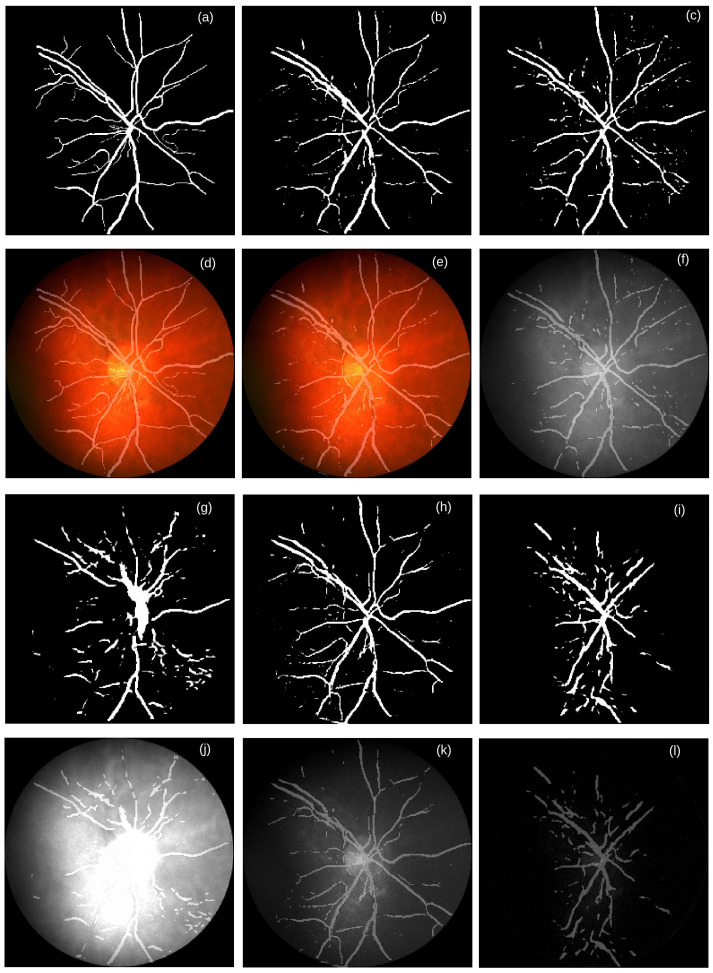
Examples of generated masks by the color-specific U-Nets for segmenting the CRBVs. The reference mask and the generated masks are shown in the first and third rows, whereas different color channels overlaid by masks are shown in the second and fourth rows. (**a**) the reference mask & (**d**) RGB fundus photograph overlaid by the reference mask, (**b**) generated mask by the U-Net trained by the RGB fundus photographs & (**e**) RGB image overlaid by the mask in (**b**), (**c**) generated mask by the U-Net trained by the grayscale fundus photographs & (**f**) Grayscaled image overlaid by the mask in (**c**), (**g**) generated mask by the U-Net trained by the red channel fundus photographs & (**j**) Red channeled fundus photograph overlaid by the mask in (**g**), (**h**) generated mask by the U-Net trained by the green channel fundus photographs & (**k**) Green channel image overlaid by the mask in (**h**), and (**i**) generated mask by the U-Net trained by the blue channel fundus photographs & (**l**) Blue channel image overlaid by the mask in (**i**). Source of image: CHASE_DB1/Image_14R.jpg.

We find that our U-Nets trained for the RGB, *gray*, and *green* channel images can segment thick vessels quite well, whereas they are in general not good at segmenting thin blood vessels. As shown in Figure 7b,e, Figure 7c,f, and Figure 7h,k, discontinuity occurs in the thin vessels segmented by our U-Nets.

The performance of U-Nets also depends to some extent on how accurately CRBVs are marked in the reference masks. Among the five data sets, the reference masks of the DRIVE data set are very accurate for both thick and thin vessels. That could be one reason we get the best performance for this data set. On the contrary, we get the worst performance for the UoA-DR data set because of the inaccurate reference masks (see Appendix F for more details). If the reference masks have inaccurate information, then the estimated performance of the U-Nets will be lower than what it should be. Two things can happen when reference masks are inaccurate. The first thing is that inaccurate reference masks in the training set may deteriorate the performance of the U-Net. However, if most reference masks are accurate enough, the deterioration may be small. The second thing is that inaccurate reference masks in the test set can generate inaccurate values for the estimated metrics. These two cases happen for the UoA-DR data set. Our U-Nets can tackle the negative effect of inaccurate reference masks in the training set of the UoA-DR. Our U-Nets learn to predict the majority of the thick vessels and some parts of thin vessels quite accurately for the UoA-DR data set. However, because of the inaccurate reference masks of the test data, the precision and recall are extremely low for all channels for the UoA-DR data set.

**Figure 8:**
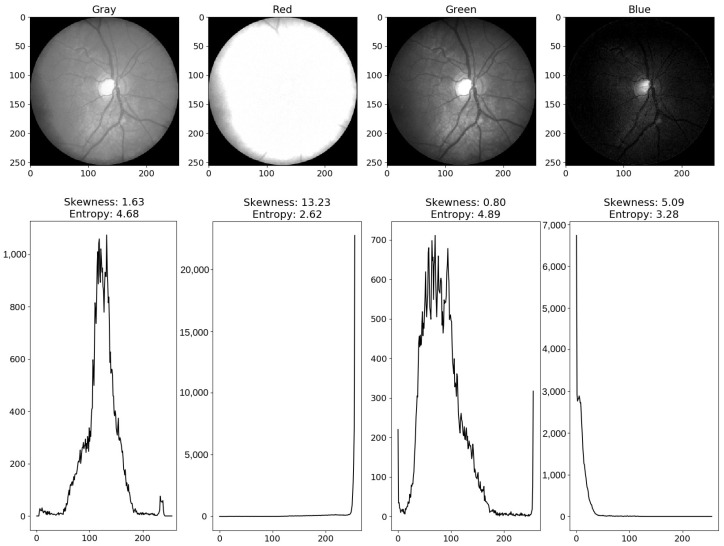
Example of overexposed *red* channel and underexposed *blue* channel of a retinal image. First row shows different channels of a fundus photograph and second row shows their corresponding histograms. Histograms of inappropriately exposed images are highly skewed and have low entropy. Source of image: CHASE_DB1/Image_11R.jpg.

We also notice that quite often, the *red* channel is affected by the overexposure, whereas the *blue* channel is affected by the underexposure (see Table 15). Both kinds of inappropriate exposure wash out retinal information that causes low entropy. Therefore, the generated masks for segmenting CRBVs do not have lines in the inappropriately exposed parts of a fundus photograph (see the overexposed part of the red channel in Figure 7j and the underexposed part of the blue channel in Figure 7l). Note that histograms of inappropriately exposed images are highly skewed and have low entropy (as shown in Figure 8).

It is not surprising that using all three color channels (i.e., RGB images) as input to the U-Net performs the best since the convolutional layers of the U-Net are flexible enough to use all information from the three color channels appropriately. By using multiple filters in each convolutional layer, U-Nets can extract multiple features from the retinal images, many of which are appropriate for segmentation. As discussed in Section 3, previous works based on non-neural network-based models usually used one color channel, most likely because these models could not be benefited from the information contained in three channels. The fact that the individual color channel performs well in certain situations raises two questions regarding the camera design:

1. Would it be worth it to develop cameras with only one color channel rather than *red*, *green*, and *blue*, possibly customized for retina analysis?
2. Could a more detailed representation of the spectrum than RGB improve the automatic analysis of retinas? The RGB representation captures the information from the spectrum that the human eye can recognize. Perhaps this is not all information from the spectrum that an automatic system could have used.

To fully answer those questions, many hardware developments would be needed. However, an initial analysis to address the first question could be to tune the weights used to produce the grayscale image from the RGB images.

## 6. Conclusions

We conduct an extensive survey to investigate which color channel in color fundus photographs is most commonly preferred for automatically diagnosing retinal diseases. We find that the *green* channel images dominate previous non-neural network-based works while all three color channels together, i.e., RGB images, dominate neural network-based works. In non-neural network-based works, researchers almost ignored the *red* and *blue* channels, reasoning that these channels are prone to poor contrast, noise, and inappropriate exposure. However, no works provided a conclusive experimental comparison of the performance of different color channels. In order to fill up that gap we conduct systematic experiments. We use a well-known U-shaped deep neural network (U-Net) to investigate which color channel is best for segmenting retinal atrophy and three retinal landmarks (i.e., central retinal blood vessels, optic disc, and macula). In our U-Net based segmentation approach, we see that segmentation of retinal landmarks and retinal atrophy can be conducted more accurately when RGB images are used than when a single channel is used. We also notice that as a single channel, the *red* channel is bad for segmenting the central retinal blood vessels, but better than other single channels for the optic disc segmentation. Although, the *blue* channel is a bad choice for segmenting the central retinal blood vessels, it is reasonably good for segmenting macula and very good for segmenting retinal atrophy. For all cases, RGB images perform the best which reveals the fact that the *red* and *blue* channels can provide supplementary information to the *green* channel. Therefore, we can conclude that all color channels are important in color fundus photographs.

## Figures and Tables

**Table 1:** Color distribution in previous works for the automatic detection of retinal diseases and segmentation of retinal landmarks and atrophy. NN: Neural network-based approaches, Non-NN: Non-neural network-based approaches.

Color	Number of Papers											
	Disease Detection						Segmentation					
	Non-NN			NN			Non-NN			NN		
	Total	Q1	Q2	Total	Q1	Q2	Total	Q1	Q2	Total	Q1	Q2
	(42)	(30)	(12)	(35)	(28)	(7)	(77)	(56)	(21)	(37)	(28)	(9)
RGB	18	9	9	29	24	5	14	10	4	28	22	6
R	7	5	2	2	1	1	15	9	6	0	0	0
G	22	11	11	4	2	2	59	43	16	10	8	2
B	3	3	0	1	1	0	8	7	1	0	0	0
Gr	6	3	3	5	4	1	7	5	2	3	0	3

**Table 2:** Color channel used in non-neural Network (Non-NN) based previous works for automatically detecting diseases in retina. DR: Diabetic Retinopathy, AMD: Age-related Macular Degeneration, DME: Diabetic Macular Edema, R: Red, G: Green, B: Blue, Gr: Grayscale weighted summation of Red, Green and Blue.

Year	Glaucoma		AMD & DME		DR	
	Reference	Color	Reference	Color	Reference	Color
2000					Hipwell [26]	G, B
2002			Walter [27]	G		
2004			Klein [28]	RGB		
2007			Scott [29]	RGB		
2008			Kose [30]	RGB	Abramoff [31]	RGB
			Gangnon [32]	RGB		
2010	Bock [33]	G	Kose [34]	Gr		
	Muramatsu [35]	R, G				
2011	Joshi [36]	R	Agurto [37]	G	Fadzil [38]	RGB
2012	Mookiah [39]	Gr	Hijazi [40]	RGB		
			Deepak [41]	RGB, G		
2013					Akram [42]	RGB
					Oh [43]	RGB
2014	Fuente-Arriaga [44]	R, G			Akram [45]	RGB
	Noronha [46]	RGB	Mookiah [47]	G	Casanova [48]	RGB
2015	Issac [49]	R, G	Mookiah [50]	R, G	Jaya [51]	RGB
	Oh [52]	G, Gr				
2016	Singh [53]	G, Gr	Acharya [54]	G	Bhaskaranand [55]	RGB
			Phan [56]	G		
			Wang [57]	RGB		
2017	Acharya [58]	Gr	Acharya [59]	G	Leontidis [60]	RGB
	Maheshwari [61]	R, G, B, Gr				
	Maheshwari [62]	G				
2018					Saha [63]	G, RGB
2020					Colomer [64]	G

**Table 3:** Color channel used in neural network (NN) based previous works for automatically detecting diseases in retina. DR: Diabetic Retinopathy, AMD: Age-related Macular Degeneration, DME: Diabetic Macular Edema, Gr: Grayscale weighted summation of Red, Green and Blue, R: Red, G: Green, B: Blue.

Year	Glaucoma		AMD & DME		DR	
	Reference	Color	Reference	Color	Reference	Color
1996					Gardner [65]	RGB
2009	Nayak [66]	R, G				
2014					Ganesan [67]	Gr
2015			Mookiah [68]	G		
2016	Asoka [69]	Gr			Abramoff [70]	RGB
					Gulshan [71]	RGB
2017	Zilly [72]	G, Gr	Burlina [73]	RGB	Abbas [74]	RGB
	Ting [75]	RGB	Burlina [76]	RGB	Gargeya [77]	RGB
					Quellec [78]	RGB
2018	Ferreira [79]	RGB, Gr	Grassmann [80]	RGB	Khojasteh [81]	RGB
	Raghavendra [82]	RGB	Burlina [83]	RGB	Lam [84]	RGB
	Li [85]	RGB				
	Fu [86]	RGB				
	Liu [87]	RGB				
2019	Liu [88]	R, G, B, Gr	Keel [89]	RGB	Li [90]	RGB
	Diaz-Pinto [91]	RGB	Peng [92]	RGB	Zeng [93]	RGB
			Matsuba [94]	RGB	Raman [95]	RGB
2020			Singh [96]	RGB		
			Gonzalez-Gonzalo [97]	RGB		
2021	Gheisari [98]	RGB				

**Table 4:** Color channel used in non-neural network (Non-NN) based previous works for segmenting retinal landmarks. OD: Optic Disc, CRBVs: Central Retinal Blood Vessels, Gr: Grayscale weighted summation of Red, Green and Blue, R: Red, G: Green, B: Blue.

Year	OD		Macula/Fovea		CRBVs	
	Reference	Color	Reference	Color	Reference	Color
1989					Chaudhuri [99]	G
1999			Sinthanayothin [100]	RGB		
2000					Hoover [10]	RGB
2004	Lowell [101]	Gr	Li [102]	RGB		
2006					Soares [103]	G
2007	Xu [104]	RGB	Niemeijer [105]	G	Ricci [106]	G
	Abramoff [107]	R, G, B	Tobin [108]	G		
2008	Youssif [109]	RGB				
2009			Niemeijer [110]	G	Cinsdikici [111]	G
2010	Welfer [112]	G				
	Aquino [113]	R, G				
	Zhu [114]	RGB				
2011	Lu [115]	R, G	Welfer [116]	G	Cheung [117]	RGB
					Kose [118]	RGB
					You [119]	G
2012					Bankhead [120]	G
			Qureshi [121]	G	Fraz [4]	G
					Fraz [122]	G
					Li [123]	RGB
					Lin [124]	G
					Moghimirad [125]	G
2013	Morales [126]	Gr	Chin [127]	RGB	Akram [128]	G
			Gegundez [129]	G	Badsha [130]	Gr
					Budai [6]	G
					Fathi [131]	G
					Fraz [132]	G
					Nayebifar [133]	G, B
					Nguyen [134]	G
					Wang [135]	G
2014	Giachetti [136]	G, Gr	Kao [137]	G	Bekkers [138]	G
			Aquino [139]	R, G	Cheng [140]	G
2015	Miri [141]	R, G, B			Dai [142]	G
	Mary [143]	R			Hassanien [144]	G
	Harangi [145]	RGB, G			Imani [146]	G
					Lazar [147]	G
					Roychowdhury [148]	G
2016	Mittapalli [149]	RGB	Medhi [150]	R	Aslani [151]	G
	Roychowdhury [152]	G	Onal [153]	Gr	Bahadarkhan [154]	G
	Sarathi [155]	R, G			Christodoulidis [156]	G
					Orlando [157]	G
2018			Ramani [158]	G	Khan [159]	G
			Chalakkal [160]	RGB	Xia [161]	G
2019	Thakur [162]	Gr			Khawaja [163]	G
	Naqvi [164]	R, G			Wang [165]	RGB
2020	Dharmawan [166]	R, G, B	Carmona [167]	G	Saroj [168]	Gr
			Guo [169]	G	Zhang [170]	G
					Zhou [171]	G
2021			Kim [172]	G		

**Table 5:** Color channel used in neural network (NN) based previous works for segmenting retinal landmarks. OD: Optic Disc, CRBVs: Central Retinal Blood Vessels, Gr: Grayscale weighted summation of Red, Green and Blue, R: Red, G: Green, B: Blue.

Year	OD		Macula/Fovea		CRBVs	
	Reference	Color	Reference	Color	Reference	Color
2011					Marin [173]	G
2015					Wang [174]	G
2016					Liskowski [175]	G
2017					Barkana [176]	G
					Mo [177]	RGB
2018	Fu [178]	RGB	Al-Bander [179]	Gr	Guo [180]	G
					Guo [181]	RGB
					Hu [182]	RGB
					Jiang [183]	RGB
					Oliveira [184]	G
					Sangeethaa [185]	G
2019	Wang [186]	RGB, Gr			Jebaseeli [187]	G
	Chakravarty [188]	RGB			Lian [189]	RGB
	Gu [190]	RGB			Noh [191]	RGB
	Tan [192]	RGB			Wang [193]	Gr
	Jiang [194]	RGB				
2020	Gao [195]	RGB			Feng [196]	G
	Jin [197]	RGB			Tamim [198]	G
	Sreng [199]	RGB				
	Bian [200]	RGB				
	Almubarak [201]	RGB				
	Tian [202]	RGB				
	Zhang [203]	RGB				
	Xie [204]	RGB				
2021	Bengani [205]	RGB	Hasan [206]	RGB	Gegundez-Arias [207]	RGB
	Veena [208]	RGB				
	Wang [209]	RGB				

**Table 6:** Color channel used for automatically detecting atrophy in retina. R: Red, G: Green, B: Blue.

Year	Non-NN		NN	
	Reference	Color	Reference	Color
2011	Lu [210]	R, B		
2012	Cheng [211]	R, G, B		
	Lu [212]	R, B		
2018	Septiarini [213]	R, G		
2020	Li [214]	R, G, B	Chai [215]	RGB
			Son [216]	RGB
2021			Sharma [217]	RGB

**Table 7:** Data sets used in our experiments.

Data Set	Height × Width	Field-of-View	Fundus Camera	Number of Images
CHASE_DB1	960×999	$30^{\circ }$	Nidek NM-200-D	28
DRIVE	584×565	$45^{\circ }$	Canon CR5-NM 3CCD	40
HRF	3264×4928	$45^{\circ }$	Canon CR-1	45
IDRiD	2848×4288	$50^{\circ }$	Kowa VX-10α	81
PALM	1444×1444 2056×2124	$45^{\circ }$	Zeiss VISUCAM 500 NM	400
STARE	605×700	$35^{\circ }$	TopCon TRV-50	20
UoA-DR	2056×2124	$45^{\circ }$	Zeiss VISUCAM 500	200

**Table 8:** Training, validation and test sets used in our experiments.

Segmentation of	Data Set	Number of Images in		
		Training Set	Validation Set	Test Set
CRBVs	CHASE_DB1	7	5	16
	DRIVE	10	8	22
	HRF	11	9	25
	STARE	5	4	11
	UoA-DR	50	40	110
Optic Disc	IDRiD	20	16	45
	PALM	100	80	220
	UoA-DR	50	40	110
Macula	PALM	100	80	220
	UoA-DR	50	40	110
Atrophy	PALM	100	80	220

**Table 9:** Performance (mean ± standard deviation) of U-Nets using different color channels for segmenting optic disc.

Color	Dataset	Precision	Recall	AUC	MIoU
RGB	IDRiD	0.897 ± 0.018	0.877 ± 0.010	0.940 ± 0.005	0.896 ± 0.003
	PALM	0.859 ± 0.009	0.862 ± 0.013	0.933 ± 0.006	0.873 ± 0.003
	UoA_DR	0.914 ± 0.012	0.868 ± 0.006	0.936 ± 0.003	0.895 ± 0.004
Gray	IDRiD	0.868 ± 0.020	0.902 ± 0.016	0.952 ± 0.007	0.892 ± 0.004
	PALM	0.758 ± 0.020	0.737 ± 0.025	0.870 ± 0.011	0.788 ± 0.009
	UoA_DR	0.907 ± 0.007	0.840 ± 0.005	0.923 ± 0.002	0.876 ± 0.008
Red	IDRiD	0.892 ± 0.006	0.872 ± 0.008	0.936 ± 0.004	0.892 ± 0.004
	PALM	0.798 ± 0.004	0.824 ± 0.012	0.912 ± 0.006	0.837 ± 0.003
	UoA_DR	0.900 ± 0.007	0.854 ± 0.006	0.928 ± 0.003	0.885 ± 0.003
Green	IDRiD	0.837 ± 0.023	0.906 ± 0.009	0.953 ± 0.004	0.882 ± 0.008
	PALM	0.708 ± 0.012	0.718 ± 0.013	0.859 ± 0.006	0.771 ± 0.004
	UoA_DR	0.895 ± 0.009	0.821 ± 0.010	0.912 ± 0.005	0.869 ± 0.006
Blue	IDRiD	0.810 ± 0.038	0.715 ± 0.011	0.858 ± 0.005	0.799 ± 0.010
	PALM	0.662 ± 0.032	0.692 ± 0.019	0.845 ± 0.009	0.748 ± 0.008
	UoA_DR	0.873 ± 0.012	0.800 ± 0.009	0.901 ± 0.004	0.851 ± 0.002

**Table 10:** Performance (mean ± standard deviation) of U-Nets using different color channels for segmenting CRBVs.

Color	Dataset	Precision	Recall	AUC	MIoU
RGB	CHASE_DB1	0.795 ± 0.005	0.638 ± 0.004	0.840 ± 0.002	0.696 ± 0.018
	DRIVE	0.851 ± 0.007	0.519 ± 0.009	0.781 ± 0.004	0.696 ± 0.013
	HRF	0.730 ± 0.017	0.633 ± 0.007	0.838 ± 0.005	0.651 ± 0.021
	STARE	0.822 ± 0.009	0.488 ± 0.010	0.766 ± 0.006	0.654 ± 0.011
	UoA_DR	0.373 ± 0.003	0.341 ± 0.008	0.669 ± 0.005	0.556 ± 0.004
Gray	CHASE_DB1	0.757 ± 0.019	0.635 ± 0.016	0.834 ± 0.009	0.648 ± 0.040
	DRIVE	0.864 ± 0.014	0.529 ± 0.014	0.786 ± 0.008	0.673 ± 0.032
	HRF	0.721 ± 0.032	0.617 ± 0.008	0.825 ± 0.005	0.605 ± 0.038
	STARE	0.810 ± 0.021	0.522 ± 0.022	0.784 ± 0.011	0.619 ± 0.031
	UoA_DR	0.373 ± 0.007	0.298 ± 0.022	0.648 ± 0.012	0.540 ± 0.009
Red	CHASE_DB1	0.507 ± 0.018	0.412 ± 0.007	0.703 ± 0.005	0.602 ± 0.001
	DRIVE	0.713 ± 0.026	0.391 ± 0.016	0.705 ± 0.010	0.637 ± 0.005
	HRF	0.535 ± 0.027	0.349 ± 0.014	0.680 ± 0.008	0.581 ± 0.004
	STARE	0.646 ± 0.040	0.271 ± 0.011	0.649 ± 0.008	0.563 ± 0.005
	UoA_DR	0.304 ± 0.011	0.254 ± 0.012	0.621 ± 0.006	0.539 ± 0.002
Green	CHASE_DB1	0.781 ± 0.017	0.676 ± 0.021	0.858 ± 0.007	0.691 ± 0.059
	DRIVE	0.862 ± 0.011	0.541 ± 0.026	0.794 ± 0.012	0.703 ± 0.047
	HRF	0.754 ± 0.018	0.662 ± 0.020	0.856 ± 0.008	0.647 ± 0.077
	STARE	0.829 ± 0.018	0.558 ± 0.028	0.806 ± 0.011	0.662 ± 0.052
	UoA_DR	0.384 ± 0.007	0.326 ± 0.023	0.662 ± 0.012	0.552 ± 0.011
Blue	CHASE_DB1	0.581 ± 0.024	0.504 ± 0.023	0.751 ± 0.010	0.638 ± 0.004
	DRIVE	0.771 ± 0.016	0.449 ± 0.015	0.736 ± 0.008	0.657 ± 0.007
	HRF	0.473 ± 0.016	0.279 ± 0.016	0.633 ± 0.007	0.558 ± 0.004
	STARE	0.446 ± 0.014	0.242 ± 0.018	0.608 ± 0.007	0.535 ± 0.003
	UoA_DR	0.316 ± 0.010	0.271 ± 0.015	0.630 ± 0.007	0.540 ± 0.002

**Table 11:** Performance (mean ± standard deviation) of U-Nets using different color channels for segmenting macula.

Color	Dataset	Precision	Recall	AUC	MIoU
RGB	PALM	0.732 ± 0.016	0.649 ± 0.029	0.825 ± 0.014	0.753 ± 0.009
	UoA_DR	0.804 ± 0.027	0.713 ± 0.043	0.858 ± 0.021	0.794 ± 0.012
Gray	PALM	0.712 ± 0.024	0.638 ± 0.016	0.819 ± 0.007	0.744 ± 0.003
	UoA_DR	0.811 ± 0.017	0.712 ± 0.018	0.858 ± 0.008	0.796 ± 0.005
Red	PALM	0.719 ± 0.013	0.648 ± 0.015	0.823 ± 0.007	0.749 ± 0.005
	UoA_DR	0.768 ± 0.006	0.726 ± 0.013	0.863 ± 0.006	0.790 ± 0.003
Green	PALM	0.685 ± 0.020	0.641 ± 0.004	0.820 ± 0.002	0.739 ± 0.005
	UoA_DR	0.791 ± 0.013	0.693 ± 0.011	0.848 ± 0.005	0.783 ± 0.005
Blue	PALM	0.676 ± 0.020	0.637 ± 0.019	0.817 ± 0.009	0.734 ± 0.002
	UoA_DR	0.801 ± 0.035	0.649 ± 0.013	0.826 ± 0.006	0.769 ± 0.012

**Table 12:** Performance (mean ± standard deviation) of U-Nets using different color channels for segmenting atrophy.

Color	Dataset	Precision	Recall	AUC	MIoU
RGB	PALM	0.719 ± 0.033	0.638 ± 0.030	0.814 ± 0.014	0.707 ± 0.019
Gray	PALM	0.630 ± 0.021	0.571 ± 0.025	0.777 ± 0.012	0.658 ± 0.039
Red	PALM	0.514 ± 0.010	0.430 ± 0.029	0.705 ± 0.013	0.596 ± 0.015
Green	PALM	0.695 ± 0.009	0.627 ± 0.032	0.808 ± 0.015	0.714 ± 0.011
Blue	PALM	0.711 ± 0.015	0.578 ± 0.016	0.785 ± 0.008	0.687 ± 0.018

**Table 13:** Number of cases where a U-Net marks OD and macula correctly in the masks. N: Total number of fundus photographs in the test set.

Segmentation for	N	Number of Cases in				
		RGB	Gray	Red	Green	Blue
Optic Disc (OD)	375	329	324	316	303	297
Macula	330	270	265	271	265	267

**Table 14:** Number of cases where a U-Net marks multiple places as OD and macula in the masks. N: Total number of fundus photographs in the test set.

Segmentation for	N	Number of Cases in				
		RGB	Gray	Red	Green	Blue
Optic Disc (OD)	375	29	26	43	46	43
Macula	330	17	25	14	17	14

**Table 15:** Number of inappropriately exposed fundus photographs. N: Total number RGB fundus photographs in the test set of a specific data set.

Data Set	N	Number of Cases in Each Color Channel							
		Where |skewness|>6.0				Where entropy<3.0			
		Gray	Red	Green	Blue	Gray	Red	Green	Blue
CHASE_DB1	28	0	10	0	13	0	4	0	3
DRIVE	40	0	12	0	0	0	1	0	3
HRF	45	0	0	0	0	0	0	0	2
IDRiD	81	0	2	0	6	0	0	0	23
PALM	400	0	0	1	40	0	0	2	121
STARE	20	0	2	0	10	0	0	0	4
UoA-DR	200	0	0	0	22	0	0	0	88

**Figure A1:**
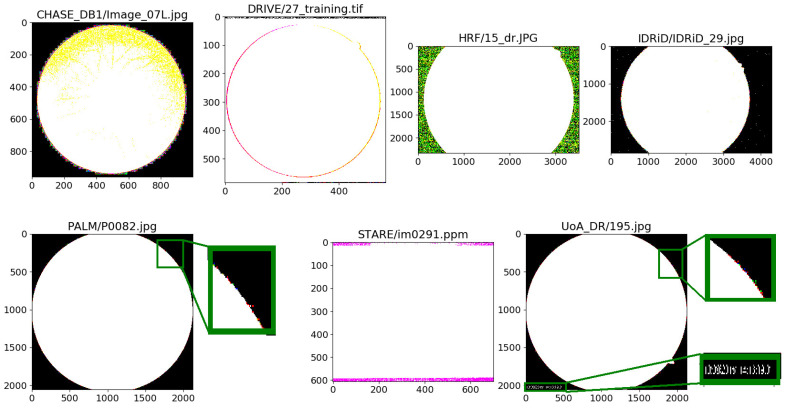
Noisy pixels in the background of the data sets we experiment on are highlighted by binary thresholding with a threshold equal to 0.

**Figure A2:**
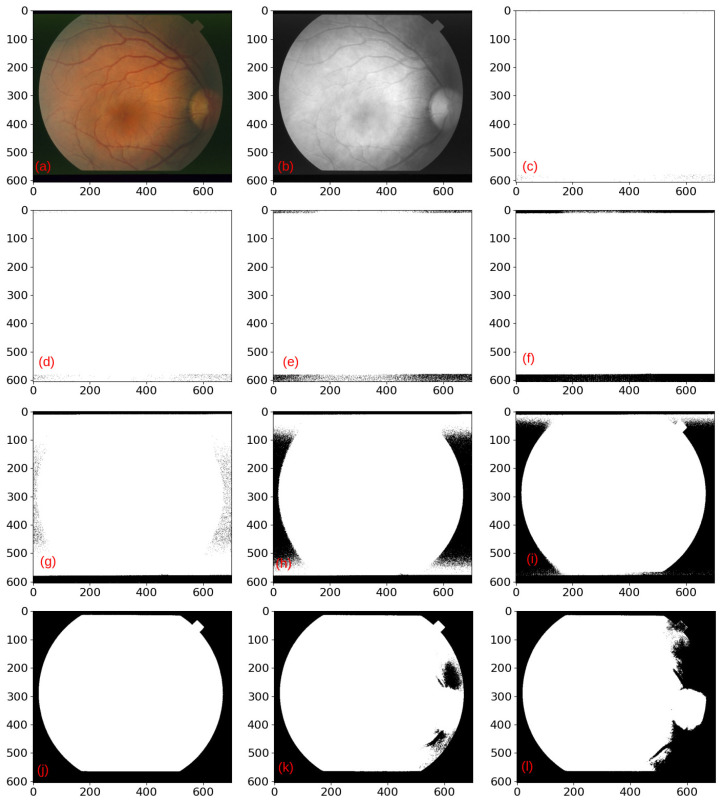
Effect of threshold value, θ, on the binary background mask when the background is noisy. (**a**) RGB image, (**b**) red channel, and (**c**–**l**) binary background masks for different θ. In (**c**) θ=0, (**d**) θ=1, (**e**) θ=5, (**f**) θ=10, (**g**) θ=15, (**h**) θ=20, (**i**) θ=25, (**j**) θ=35, (**k**) θ=55, and (**l**) θ=65. Depending on the intensity of the background noise, we need to decide the value of θ. If we assign too small value to θ, then some noisy background pixels may be considered as the foreground (see (**c**–**i**)). If we set too high value to θ, then some area of the foreground will be considered as the background (see (**k**,**l**)). Source of fundus photograph: STARE data set and image file: im0240.ppm.

**Figure A3:**
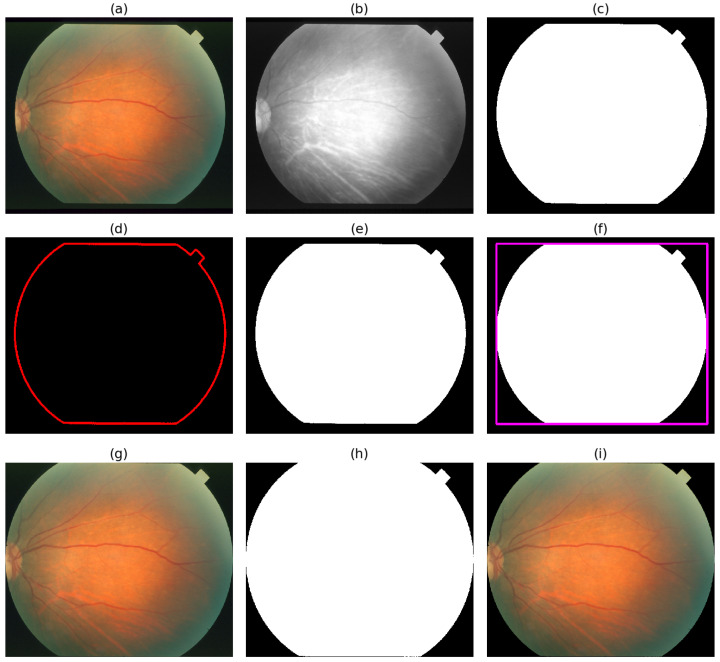
Steps of cropping unnecessary background and removing background noise of a colored fundus photograph. (**a**) RGB fundus photograph [Source of fundus photograph: STARE data set and image file: im0291.ppm.], (**b**) Red channel of the fundus photograph, (**c**) Background mask generated by global thresholding using θ=35, (**d**) Boundary contour, (**e**) Background mask generated by filling boundary contour, (**f**) Background mask with minimum bounding rectangle (MBR), (**g**) Cropped RGB fundus photograph generated by using the width, height, and position of the MBR, (**h**) Cropped background mask generated by using the width, height and position of the MBR, and (**i**) Cropped RGB fundus photograph after removing background noise by using cropped background mask.

**Figure A4:**
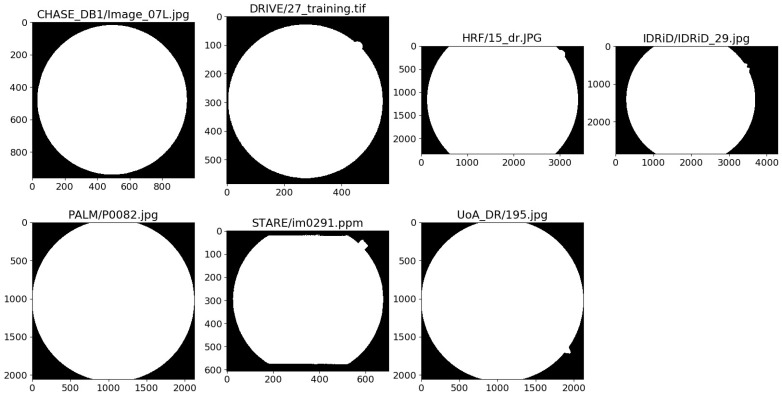
Generated foreground masks of seven fundus photographs of seven data sets.

**Figure A5:**
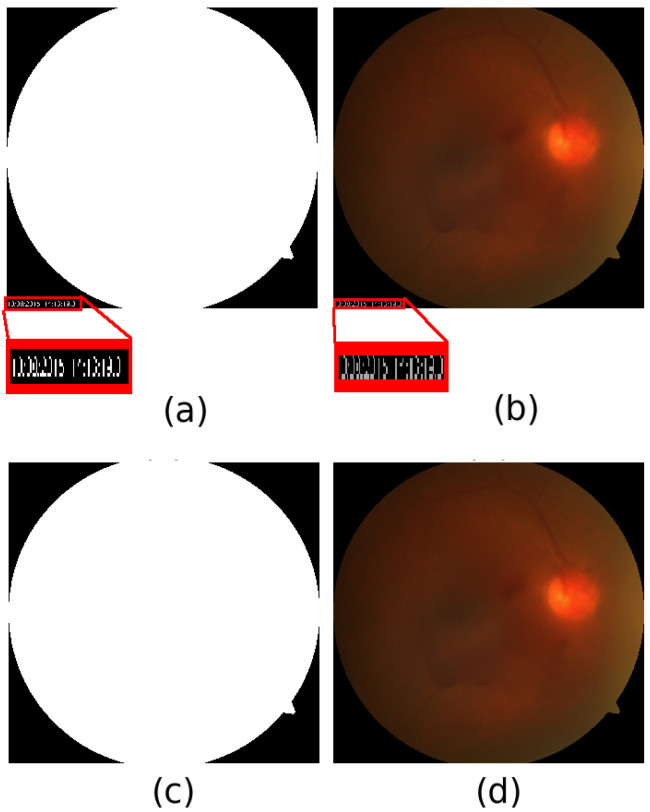
Effect of background masks, $B_{1}$ and $B_{2}$ on an RGB fundus photograph. (**a**) background mask, $B_{1}$, generated by global binary thresholding, (**b**) retinal image masked by $B_{1}$, (**c**) background mask, $B_{2}$, generated by filling the boundary contour, and (**d**) retinal image masked by $B_{2}$. When intensity of background noise is very high, global thresholding cannot generate $B_{1}$ with zero values for the background pixels. Therefore, the masked image is not noise free. On the other hand, $B_{2}$ is background noise free and so the masked image. Source of fundus photograph: UoA-DR data set and image file: 195.jpg.

**Figure A6:**
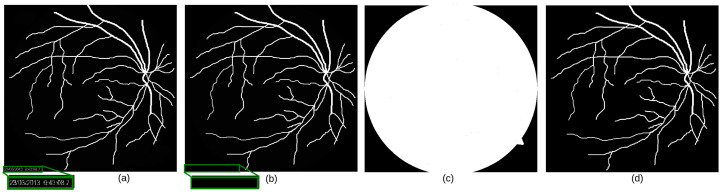
Effect of binarization on provided mask for segmenting CRBVs. (**a**) Provided mask, (**b**) Provided mask after removing the background noise outside the *field-of-view* (FOV) area but having invisible background noise inside the FOV area, (**c**) Binary mask after highlighting invisible background noise inside the FOV area by global binary thresholding using θ=0, and (**d**) Binary mask generated by global binary thresholding using θ=0 for segmenting CRBVs.

**Figure A7:**
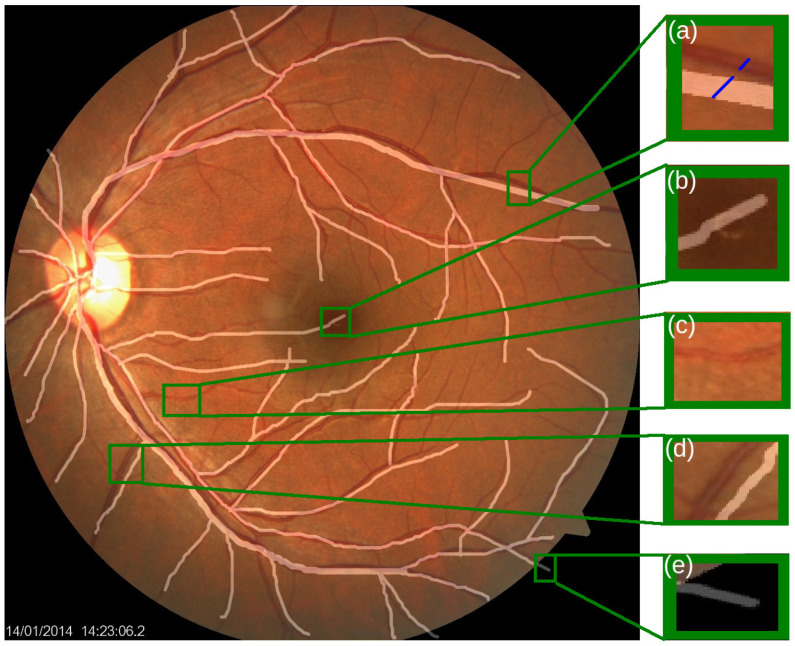
A fundus photograph of the UoA-DR data set overlaid by the reference binary mask. Rectangles point out some inaccurately marked places with the green border. (**a**) Mismatched thickness between the real blood vessels (dark red colored) and marked line (white-colored), (**b**) Inaccurate marked line (white-colored) gone to the Foveal Avascular Zone (FAZ), which is a region in the center of the macula that is entirely lacking in CRBVs, (**c**) Unmarked clearly visible CRBV, (**d**) Inaccurate marked line (white-colored) whose position is different from the original vessel (dark red colored), and (**e**) Inaccurate marked line which goes to the background. Source of image: UoA-DR data set and image file: 163.jpg.

## Data Availability

All data sets used in this work are publicly available as described in Section 4.2.

## Appendix A. Generating Background Mask

The background of a fundus photograph can be noisy, i.e., the background pixels can have non-zero values. Noisy background pixels, in general, are invisible to the bare eye because of their low intensity. Exceptions to this occur. For example, images in the STARE data set have visible background noise. Moreover, sometimes non-retinal information, such as image capturing date-time and patient’s name, can be present with high intensities in the background (e.g., images in the UoA-DR data set). This kind of information is also considered as noise when they are not useful for any decision. No matter whether noise in the background is visible or invisible to human eyes, or whether the intensity of background pixels are high or low, by global binary thresholding with threshold, θ=0, we detect the presence of noisy background pixels in almost all data sets as shown in Figure A1.

Using a background mask, we can get rid of background noise. A simple method for creating a background mask would be to consider all pixels with an intensity lower than or equal to a threshold, θ, to be part of the background and the other pixels to be part of the foreground. When the image is noiseless, setting θ=0 (i.e., keeping zero-valued pixels unchanged while setting pixels with non-zero intensities to $2^{n}-1$) is good enough to generate the background mask. However, for a noisy background, if we set the threshold, θ, to a very small value (i.e., a value lower than the intensities of noise), then the background mask will consider the background parts as a foreground, as shown in Figure A2c–i. On the other hand, if we set a very high value to θ (i.e., a value higher than the intensities of foreground pixels), then some parts of the foreground may get lost in the background mask, as shown in Figure A2k,l. Of course, in reality, some background pixels may have a higher intensity than some foreground pixels so that no threshold would accurately separate the foreground from the background. Further, the optimal threshold may depend on the data set.

As a more robust procedure for generating background masks for removing background noise, we apply the following steps:

- Step-1: Generate a preliminary background mask, $B_{1}$, by global binary thresholding, i.e., by setting the pixel intensity, *p*, of a single channeled image, *I*, to 0 or $2^{n}-1$ in the following way:
  $$p=\left\{\begin{array}{cc} 0 & \;\mathrm{if}\;p\leq \theta \\ 2^{n}-1 & \;\mathrm{if}\;p>\theta , \end{array}\right.$$
  where *n* is the number of bit used for the intensity of *p* (see Figure A3c). For an 8-bit image, $2^{n}-1=255$. Note that we are using the red channeled image, $I_{R}$. By trial-and-error, we finally set θ to 15, 40, 35, 35, 5, 35, and 5 to get good preliminary background masks for the CHASE_DB1, DRIVE, HRF, IDRiD, PALM, STARE, and UoA-DR data sets, respectively.
- Step-2: Determine the boundary contour of the retina by finding the contour which has the maximum area. Note that a contour is a closed curve joining all the straight points having the same color or intensity (see Figure A3d).
- Step-3: Set the pixels inside the boundary contour to $2^{n}-1$ and outside the boundary contour to zero in order to generate the final background mask, $B_{2}$ (see Figure A3e).

Figure A4 shows seven examples of generated binary background masks and Figure A5 illustrates the benefit of using $B_{2}$ instead of $B_{1}$ for masking out the high-intensity background noise caused by text information in an image.

Using the provided masks of the DRIVE and HRF data sets, we estimate the performance of our approach of generating binary background masks. As shown in Table A1, our approach is highly successful.

**Table A1:** Performance of our approach of generating background masks.

Data Set	Precision	Recall	AUC	MIoU
DRIVE	0.997	0.997	0.996	0.995
HRF	1.000	1.000	1.000	1.000

## Appendix B. Cropping Out Background

The background of an image, $I_{x}$, does not contain any information about the retina, which can be helpful for automatic retina-related tasks. Note that $I_{x}$ can be an RGB image, a single channeled image, or a binary mask for segmenting OD, macula, CRBVs, or retinal atrophy. As a robust procedure for cropping the unnecessary background and removing background noise from the $I_{x}$, we apply the following steps:

- Step-1: Generate the background mask, $B_{2}$, using the steps described in Appendix A.
- Step-2: Determine the minimum bounding rectangle (MBR) which minimally covers the background mask, $B_{2}$ (See Figure A3f).
- Step-3: Crop $I_{x}$ and $B_{2}$ equal to the MBR (see Figure A3g,h).
- Step-4: Remove background noise from the cropped $I_{x}$ by masking it by the cropped $B_{2}$ (see Figure A3i).

## Appendix C. Turning Provided Reference Masks into Binary Masks

Although reference masks used for segmentation need to be binary masks (i.e., having only two pixel intensities, e.g., zero for the background pixels and 255 for the foreground pixels of an 8-bit image), we notice that two data sets (i.e., HRF and UoA-DR) do not fulfill this requirement, as shown in Table A2. Three out of 45 provided masks of the HRF data set, and all 200 provided masks of the UoA-DR data set have pixels of multiple intensities. There are two cases: the first case is that the noisy background pixels which are supposed to be 0 have intensities other than zero and the second case is that the foreground pixels, which are supposed to be 255 have intensities other than 255. We also notice that even though the provided masks of the IDRiD data set are binary masks, however, the maximum intensity is 29 instead of 255.

We turn all provided masks into binary masks having pixel intensity [0,255] by global binary thresholding with threshold, θ=127. Before binarization, we remove noisy pixels from the outside of the *field-of-view* area by using the estimated background mask, $B_{2}$ (see Figure A6b for an example). As shown in Figure A6c, there will still be noisy pixels inside the FOV area. For that, we apply binary thresholding and generate the final binary mask as shown in Figure A6d.

**Table A2:** Distribution of provided binary and non-binary masks for segmenting CRBVs, optic discs, macula and retinal atrophy. n: total number of provided masks, m: number of provided binary masks.

Segmentation Type	CHASE_DB1		DRIVE		HRF		IDRiD		PALM		STARE		UoA-DR	
	n	m	n	m	n	m	n	m	n	m	n	m	n	m
CRBVs	28	0	40	0	45	0	0	0	0	0	40	0	200	200
Optic Disc	0	0	0	0	0	0	81	0	400	0	0	0	200	200
Macula	0	0	0	0	0	0	0	0	0	0	0	0	0	0
Retinal Atrophy	0	0	0	0	0	0	0	0	311	0	0	0	0	0

## Appendix D. Generating Binary Masks for Segmenting Macula

Even though three data sets (i.e., IDRiD, PALM, and UoA-DR) provide reference masks for segmenting the optic disc (OD), five data sets (i.e., CHASE_DB1, DRIVE, HRF, STARE, and UoA-DR) for CRBVs and one data set (i.e., PALM) for retinal atrophy, none of the seven data sets provide reference masks for segmenting macula. However, two data sets (PALM and UoA-DR) provide the center of the macula. The average size of the macula in humans is around 5.5 mm. However, the average clinical size of the macula in humans is 1.5 mm, whereas the average size of the OD is 1.825 mm (vertically 1.88 mm and horizontally 1.77 mm). We assume that the size of maculas are equal to the ODs and using the provided center values we generate binary masks for segmenting the macula using the following steps:

- Step-1: Get the corresponding reference mask, $R_{OD}$ of a color fundus photograph for segmenting OD.
- Step-2: Generate the background mask, $B_{2}$, by following the steps described in Appendix A.
- Step-3: Remove the background noise outside the foreground of $R_{OD}$ by masking it by $B_{2}$.
- Step-4: Turn $R_{OD}$ into a binary mask $R_{OD\_Binary}$ by global thresholding.
- Step-5: Find the boundary contour of the foreground of $R_{OD\_Binary}$.
- Step-6: Determine radius, *r* of the minimum closing circle of $R_{OD\_Binary}$.
- Step-7: Draw a circle in the provided center of the macula having radius *r*.
- Step-8: Set the pixels inside the circle to $2^{n}-1$ and outside the circle to 0 in order to generate the final reference mask, $R_{Macula\_Binary}$.

## Appendix E. Architecture of U-Net

Our color specific U-Nets have the architecture shown in Table A3. Similar to the original U-Net proposed in [17], our U-Nets consist of two parts: a contracting side and an expansive side. None of these sides have any fully connected layers instead both sides have mainly convolutional layers. Unlike the original U-Net, we use convolutional layer with stride two instead of a max poling layer for down-sampling in the contracting side. Instead of using unpadded convolutions we use *same* padding convolutions in both the contracting side and expansive side. Note that in the *same* padding the output size is the same as the input size. Therefore, we do not need cropping in the expansive side which was needed in the original work due to the loss of border pixels in every convolution. We use Exponential Linear Unit (ELU) instead of Rectified Linear Unit (ReLU) as activation function in each convolutional layer except the output layer. In the output layer, we usethe *sigmoid* function as the activation function. An alternative would have been the *softmax* function with two outputs. In both the contracting and expansive sides, the two padded convolutional layers are separated by a *drop-out* layer. We use a drop-out layer in order to avoid over-fitting. There are 23 convolutional layers in the original U-Net, whereas in our U-Nets there are 29 convolutional layers. In the original U-Net, there are four down-sampling blocks in the contracting side and four up-sampling blocks in the expansive side, whereas in our U-Nets there are five down-sampling and five up-sampling blocks. In total, each of our U-Nets has 5,939,521 trainable parameters.

**Table A3:** Architecture of our U-Net. #Params: Number of parameters.

Layer	Output Shape	# Params
Input	(256, 256, 1)	0
Convolution (strides = (1, 1), filters = 16, kernel = (3, 3), activation = ELU)	(256, 256, 16)	160
Dropout (0.1)	(256, 256, 16)	0
Convolution (strides = (1, 1), filters = 16, kernel = (3, 3), activation = ELU, name = **C1**)	(256, 256, 16)	2320
Convolution (strides = (2, 2), filters = 16, kernel = (3, 3), activation = ELU)	(128, 128, 16)	2320
Convolution (strides = (1, 1), filters = 32, kernel = (3, 3), activation = ELU)	(128, 128, 32)	4640
Dropout (0.1)	(128, 128, 32)	0
Convolution (strides = (1, 1), filters = 32, kernel = (3, 3), activation = ELU, name = **C2**)	(128, 128, 32)	9248
Convolution (strides = (2, 2), filters = 32, kernel = (3, 3), activation = ELU)	(64, 64, 32)	9248
Convolution (strides = (1, 1), filters = 64, kernel = (3, 3), activation = ELU)	(64, 64, 64)	18,496
Dropout (0.2)	(64, 64, 64)	0
Convolution (strides = (1, 1), filters = 64, kernel = (3, 3), activation = ELU, name = **C3**)	(64, 64, 64)	36,928
Convolution (strides = (2, 2), filters = 64, kernel = (3, 3), activation = ELU)	(32, 32, 64)	36,928
Convolution (strides = (1, 1), filters = 128, kernel = (3, 3), activation = ELU)	(32, 32, 128)	73,856
Dropout (0.2)	(32, 32, 128)	0
Convolution (strides = (1, 1), filters = 128, kernel = (3, 3), activation = ELU, name = **C4**)	(32, 32, 128)	147,584
Convolution (strides = (2, 2), filters = 128, kernel = (3, 3), activation = ELU)	(16, 16, 128)	147,584
Convolution (strides = (1, 1), filters = 256, kernel = (3, 3), activation = ELU)	(16, 16, 256)	295,168
Dropout (0.3)	(16, 16, 256)	0
Convolution (strides = (1, 1), filters = 256, kernel = (3, 3), activation = ELU, name = **C5**)	(16, 16, 256)	590,080
Convolution (strides = (2, 2), filters = 256, kernel = (3, 3), activation = ELU)	(8, 8, 256)	590,080
Convolution (strides = (1, 1), filters = 256, kernel = (3, 3), activation = ELU)	(8, 8, 256)	590,080
Dropout (0.3)	(8, 8, 256)	0
Convolution (strides = (1, 1), filters = 256, kernel = (3, 3), activation = ELU)	(8, 8, 256)	590,080
Transposed Convolution (strides = (2, 2), filters = 256, kernel = (2, 2), activation = ELU, name = **U1**)	(16, 16, 256)	262,400
Concatenation (**C5, U1**)	(16, 16, 512)	0
Convolution (strides = (1, 1), filters = 256, kernel = (3, 3), activation = ELU)	(16, 16, 256)	1,179,904
Dropout (0.3)	(16, 16, 256)	0
Convolution (strides = (1, 1), filters = 256, kernel = (3, 3), activation = ELU)	(16, 16, 256)	590,080
Transposed Convolution (strides = (2, 2), filters = 128, kernel = (2, 2), activation = ELU, name = **U2**)	(32, 32, 128)	131,200
Concatenation (**C4, U2**)	(32, 32, 256)	0
Convolution (strides = (1, 1), filters = 128, kernel = (3, 3), activation = ELU)	(32, 32, 128)	295,040
Dropout (0.2)	(32, 32, 128)	0
Convolution (strides = (1, 1), filters = 128, kernel = (3, 3), activation = ELU)	(32, 32, 128)	147,584
Transposed Convolution (strides = (2, 2), filters = 64, kernel = (2, 2), activation = ELU, name = **U3**)	(64, 64, 64)	32,832
Concatenation (**C3, U3**)	(64, 64, 128)	0
Convolution (strides = (1, 1), filters = 64, kernel = (3, 3), activation = ELU)	(64, 64, 64)	73,792
Dropout (0.2)	(64, 64, 64)	0
Convolution (strides = (1, 1), filters = 64, kernel = (3, 3), activation = ELU)	(64, 64, 64)	36,928
Transposed Convolution (strides = (2, 2), filters = 32, kernel = (2, 2), activation = ELU, name = **U4**)	(128, 128, 32)	8224
Concatenation (**C2, U4**)	(128, 128, 64)	0
Convolution (strides = (1, 1), filters = 32, kernel = (3, 3), activation = ELU)	(128, 128, 32)	18,464
Dropout (0.1)	(128, 128, 32)	0
Convolution (strides = (1, 1), filters = 32, kernel = (3, 3), activation = ELU)	(128, 128, 32)	9248
Transposed Convolution (strides = (2, 2), filters = 16, kernel = (2, 2), activation = ELU, name = **U5**)	(256, 256, 16)	2064
Concatenation (**C1, U5**)	(256, 256, 16)	0
Convolution (strides = (1, 1), filters = 16, kernel = (3, 3), activation = ELU)	(256, 256, 16)	4624
Dropout (0.1)	(256, 256, 16)	0
Convolution (strides = (1, 1), filters = 16, kernel = (3, 3), activation = ELU)	(256, 256, 16)	2320
Convolution (strides = (1, 1), filters = 1, kernel = (1, 1), activation = Sigmoid, name = Output)	(256, 256, 1)	17

## Appendix F. Inaccurate Masks in UoA_DR for Segmenting CRBVs

Among the five data sets we experiment on, the UoA-DR data set has the largest number of masks for segmenting CRBVs. Even though it could be a good data set for training and testing U-Nets, the performance of any color-specific U-Net for the UoA-DR test set is the worst among all data sets regardless of whether U-Nets are trained by combining data of five data sets together or by using data only from the UoA-DR data set. The reason behind it is that all the reference masks provided by the UoA-DR data set for segmenting CRBVs are inaccurate. In the UoA-DR data set, the reference masks usually do not match the real blood vessels well. In many places of the reference masks, vessels are marked in the wrong places. Moreover, thick vessels are marked by thinner lines, and thin vessels are marked by thicker lines in many places of reference masks. Even in some reference masks, clearly visible thin vessels are not marked as shown in Figure A7.

## Appendix G. Performance of U-Nets Trained and Tested on Individual Data Set

Since different fundus cameras capture retinal images of different data sets in different experimental setups, different data sets may be of different difficulties. We, therefore, do experiments on the different sets individually, i.e., training and testing on the same set for segmenting CRBVs. Table A4 and Table A5 show the results for CRBVs’ segmentation of five data sets: CHASE_DB1, DRIVE, HRF, STARE, and UoA_DR. The first and second blocks in these tables show the results of the U-Nets for which 25% of the data is used for training, whereas the third block shows the results of the U-Nets for which 55% of the data is used for training. In the first block, 55% of the data is sued for testing, whereas in the second and third blocks, only 25% of the data is used for testing. For all three cases, 20% of the data is used as the validation set. It should be noted that individual test sets prepared by taking 25% of the data are fairly small, so these results may not be very reliable. However, the results in the first and second blocks are fairly similar, which indicates that the results are reasonably stable. Overall, we see a substantial improvement in the third block compared to the second, suggesting that the U-Nets benefit from more training data. We also notice that both in Table 10 (same training data for all sets) and Table A4 (set specific training data), there is a large difference in the results for the different data sets which indicates that different data sets have different levels of difficulty.

**Table A4:** Effect of different amounts of training data on the performance (mean ± standard deviation) of U-Nets trained using different color channels for segmenting CRBVs. Note that the CLAHE is applied in the pre-processing stage.

CHASE_DB1					
**Data Split**	**Color**	**Precision**	**Recall**	**AUC**	**MIoU**
25% Training, 20% Validation, 55% Test	RGB	0.569 ± 0.203	0.448 ± 0.041	0.729 ± 0.059	0.537 ± 0.046
	GRAY	0.615 ± 0.081	0.412 ± 0.041	0.735 ± 0.024	0.503 ± 0.051
	RED	0.230 ± 0.030	0.332 ± 0.053	0.613 ± 0.010	0.474 ± 0.006
	GREEN	0.782 ± 0.026	0.526 ± 0.020	0.792 ± 0.007	0.606 ± 0.045
	BLUE	0.451 ± 0.114	0.370 ± 0.018	0.683 ± 0.032	0.485 ± 0.008
25% Training, 20% Validation, 25% Test	RGB	0.571 ± 0.207	0.441 ± 0.045	0.724 ± 0.062	0.538 ± 0.048
	GRAY	0.624 ± 0.080	0.407 ± 0.036	0.731 ± 0.023	0.502 ± 0.050
	RED	0.244 ± 0.037	0.342 ± 0.046	0.619 ± 0.009	0.474 ± 0.007
	GREEN	0.791 ± 0.026	0.515 ± 0.022	0.787 ± 0.008	0.602 ± 0.044
	BLUE	0.449 ± 0.116	0.362 ± 0.017	0.677 ± 0.033	0.484 ± 0.008
55% Training, 20% Validation, 25% Test	RGB	0.816 ± 0.012	0.541 ± 0.024	0.784 ± 0.012	0.684 ± 0.018
	GRAY	0.803 ± 0.002	0.515 ± 0.026	0.775 ± 0.010	0.671 ± 0.016
	RED	0.389 ± 0.039	0.363 ± 0.027	0.680 ± 0.021	0.504 ± 0.028
	GREEN	0.838 ± 0.005	0.583 ± 0.017	0.806 ± 0.009	0.687 ± 0.038
	BLUE	0.648 ± 0.019	0.383 ± 0.012	0.698 ± 0.006	0.601 ± 0.010
**DRIVE**					
**Data Split**	**Color**	**Precision**	**Recall**	**AUC**	**MIoU**
25% Training, 20% Validation, 55% Test	RGB	0.796 ± 0.036	0.443 ± 0.065	0.749 ± 0.028	0.622 ± 0.072
	GRAY	0.835 ± 0.016	0.419 ± 0.022	0.739 ± 0.009	0.590 ± 0.066
	RED	0.362 ± 0.098	0.342 ± 0.072	0.628 ± 0.015	0.476 ± 0.007
	GREEN	0.846 ± 0.010	0.463 ± 0.025	0.758 ± 0.009	0.671 ± 0.027
	BLUE	0.537 ± 0.078	0.297 ± 0.028	0.660 ± 0.022	0.512 ± 0.026
25% Training, 20% Validation, 25% Test	RGB	0.839 ± 0.035	0.442 ± 0.068	0.749 ± 0.030	0.626 ± 0.073
	GRAY	0.874 ± 0.018	0.413 ± 0.023	0.737 ± 0.009	0.592 ± 0.068
	RED	0.400 ± 0.108	0.352 ± 0.073	0.637 ± 0.014	0.476 ± 0.009
	GREEN	0.896 ± 0.009	0.462 ± 0.025	0.760 ± 0.009	0.676 ± 0.028
	BLUE	0.575 ± 0.080	0.300 ± 0.024	0.663 ± 0.020	0.512 ± 0.027
55% Training, 20% Validation, 25% Test	RGB	0.896 ± 0.005	0.539 ± 0.010	0.787 ± 0.006	0.732 ± 0.014
	GRAY	0.895 ± 0.004	0.528 ± 0.012	0.781 ± 0.005	0.731 ± 0.006
	RED	0.660 ± 0.085	0.316 ± 0.037	0.674 ± 0.017	0.520 ± 0.038
	GREEN	0.904 ± 0.003	0.533 ± 0.008	0.786 ± 0.003	0.718 ± 0.024
	BLUE	0.783 ± 0.042	0.386 ± 0.044	0.705 ± 0.021	0.645 ± 0.037
**HRF**					
**Data Split**	**Color**	**Precision**	**Recall**	**AUC**	**MIoU**
25% Training, 20% Validation, 55% Test	RGB	0.792 ± 0.006	0.537 ± 0.021	0.799 ± 0.013	0.597 ± 0.024
	GRAY	0.776 ± 0.004	0.497 ± 0.017	0.781 ± 0.011	0.579 ± 0.025
	RED	0.204 ± 0.024	0.258 ± 0.017	0.591 ± 0.014	0.467 ± 0.002
	GREEN	0.821 ± 0.013	0.578 ± 0.012	0.824 ± 0.006	0.624 ± 0.037
	BLUE	0.155 ± 0.002	0.361 ± 0.010	0.580 ± 0.001	0.482 ± 0.008
25% Training, 20% Validation, 25% Test	RGB	0.759 ± 0.006	0.535 ± 0.023	0.797 ± 0.014	0.593 ± 0.023
	GRAY	0.741 ± 0.005	0.503 ± 0.017	0.782 ± 0.011	0.576 ± 0.025
	RED	0.197 ± 0.021	0.245 ± 0.017	0.586 ± 0.013	0.467 ± 0.002
	GREEN	0.794 ± 0.016	0.581 ± 0.013	0.824 ± 0.006	0.619 ± 0.036
	BLUE	0.149 ± 0.004	0.368 ± 0.013	0.578 ± 0.002	0.480 ± 0.007
55% Training, 20% Validation, 25% Test	RGB	0.781 ± 0.008	0.608 ± 0.005	0.824 ± 0.004	0.693 ± 0.013
	GRAY	0.768 ± 0.010	0.573 ± 0.017	0.807 ± 0.009	0.677 ± 0.022
	RED	0.512 ± 0.009	0.271 ± 0.021	0.641 ± 0.013	0.536 ± 0.011
	GREEN	0.788 ± 0.006	0.647 ± 0.009	0.846 ± 0.003	0.674 ± 0.060
	BLUE	0.274 ± 0.110	0.341 ± 0.047	0.620 ± 0.032	0.500 ± 0.019
**STARE**					
**Data Split**	**Color**	**Precision**	**Recall**	**AUC**	**MIoU**
25% Training, 20% Validation, 55% Test	RGB	0.556 ± 0.204	0.300 ± 0.073	0.659 ± 0.073	0.478 ± 0.008
	GRAY	0.619 ± 0.050	0.283 ± 0.058	0.680 ± 0.033	0.478 ± 0.017
	RED	0.148 ± 0.003	0.222 ± 0.033	0.516 ± 0.009	0.468 ± 0.000
	GREEN	0.600 ± 0.242	0.351 ± 0.030	0.680 ± 0.082	0.483 ± 0.019
	BLUE	0.167 ± 0.036	0.145 ± 0.034	0.518 ± 0.021	0.469 ± 0.001
25% Training, 20% Validation, 25% Test	RGB	0.531 ± 0.195	0.334 ± 0.082	0.672 ± 0.082	0.482 ± 0.009
	GRAY	0.607 ± 0.055	0.314 ± 0.066	0.691 ± 0.039	0.483 ± 0.020
	RED	0.143 ± 0.003	0.231 ± 0.038	0.512 ± 0.011	0.471 ± 0.000
	GREEN	0.587 ± 0.243	0.376 ± 0.048	0.688 ± 0.092	0.488 ± 0.024
	BLUE	0.164 ± 0.032	0.142 ± 0.039	0.517 ± 0.020	0.472 ± 0.001
55% Training, 20% Validation, 25% Test	RGB	0.756 ± 0.014	0.448 ± 0.031	0.749 ± 0.015	0.610 ± 0.038
	GRAY	0.748 ± 0.010	0.504 ± 0.026	0.770 ± 0.010	0.656 ± 0.017
	RED	0.181 ± 0.020	0.293 ± 0.069	0.558 ± 0.008	0.474 ± 0.006
	GREEN	0.749 ± 0.013	0.550 ± 0.025	0.795 ± 0.012	0.659 ± 0.038
	BLUE	0.163 ± 0.007	0.324 ± 0.059	0.547 ± 0.006	0.469 ± 0.004
**UoA_DR**					
**Data Split**	**Color**	**Precision**	**Recall**	**AUC**	**MIoU**
25% Training, 20% Validation, 55% Test	RGB	0.320 ± 0.011	0.398 ± 0.008	0.699 ± 0.006	0.541 ± 0.015
	GRAY	0.315 ± 0.011	0.353 ± 0.016	0.675 ± 0.007	0.526 ± 0.017
	RED	0.203 ± 0.013	0.260 ± 0.016	0.614 ± 0.006	0.516 ± 0.005
	GREEN	0.332 ± 0.007	0.415 ± 0.018	0.705 ± 0.009	0.534 ± 0.014
	BLUE	0.237 ± 0.012	0.260 ± 0.008	0.620 ± 0.007	0.526 ± 0.006
25% Training, 20% Validation, 25% Test	RGB	0.313 ± 0.011	0.395 ± 0.008	0.697 ± 0.005	0.540 ± 0.015
	GRAY	0.306 ± 0.011	0.350 ± 0.016	0.673 ± 0.008	0.524 ± 0.017
	RED	0.201 ± 0.013	0.259 ± 0.015	0.614 ± 0.006	0.516 ± 0.005
	GREEN	0.326 ± 0.007	0.412 ± 0.017	0.704 ± 0.009	0.532 ± 0.014
	BLUE	0.232 ± 0.011	0.257 ± 0.007	0.618 ± 0.006	0.524 ± 0.005
55% Training, 20% Validation, 25% Test	RGB	0.333 ± 0.005	0.445 ± 0.012	0.717 ± 0.004	0.557 ± 0.007
	GRAY	0.330 ± 0.003	0.413 ± 0.014	0.700 ± 0.006	0.559 ± 0.004
	RED	0.289 ± 0.011	0.299 ± 0.007	0.641 ± 0.004	0.543 ± 0.003
	GREEN	0.335 ± 0.002	0.470 ± 0.010	0.728 ± 0.004	0.564 ± 0.004
	BLUE	0.281 ± 0.012	0.280 ± 0.013	0.630 ± 0.006	0.540 ± 0.004

## Appendix H. Effect of CLAHE

Different data sets have different qualities, which cause different levels of difficulty. One reason for poor-quality images is inappropriate contrast. In general, histogram equalization techniques such as Contrast Limited Adaptive Histogram Equalization (CLAHE) are applied to enhance the local contrast of fundus photographs. In this work, we also apply CLAHE in the pre-processing stage of the experiments mentioned above. In order to investigate the effect of CLAHE on different data sets, we conduct experiments using the fundus photographs without applying CLAHE. Table A5 shows results when CLAHE is not applied. These results are obtained using the same training/validation/test splits as in the third blocks of Table A4. Overall, CLAHE improves the results of the STARE set a lot, and also quite a lot of the DRIVE and HRF data sets. The results of the CHASE_DB1 data set are a bit mixed depending on the metric. For the UoA-DR data set, CLAHE does not seem to help at all.

**Table A5:** Performance (mean ± standard deviation) of U-Nets trained using different color channels for segmenting CRBVs when CLAHE is not applied on the retinal images in the pre-processing stage. Note that 55% data was used for training, whereas, 25% data is for validation and 25% data for testing.

Database	Color	Precision	Recall	AUC	MIoU
CHASEDB1	RGB	0.676 ± 0.057	0.419 ± 0.037	0.727 ± 0.020	0.576 ± 0.051
	GRAY	0.629 ± 0.078	0.406 ± 0.052	0.714 ± 0.025	0.570 ± 0.060
	RED	0.217 ± 0.012	0.353 ± 0.026	0.611 ± 0.006	0.476 ± 0.009
	GREEN	0.802 ± 0.017	0.530 ± 0.019	0.781 ± 0.009	0.672 ± 0.023
	BLUE	0.589 ± 0.023	0.373 ± 0.016	0.690 ± 0.006	0.556 ± 0.050
DRIVE	RGB	0.856 ± 0.024	0.470 ± 0.017	0.750 ± 0.010	0.693 ± 0.011
	GRAY	0.855 ± 0.021	0.464 ± 0.030	0.746 ± 0.015	0.693 ± 0.024
	RED	0.297 ± 0.009	0.376 ± 0.017	0.619 ± 0.003	0.472 ± 0.010
	GREEN	0.886 ± 0.006	0.509 ± 0.010	0.771 ± 0.005	0.722 ± 0.004
	BLUE	0.504 ± 0.171	0.331 ± 0.043	0.642 ± 0.031	0.551 ± 0.071
HRF	RGB	0.757 ± 0.014	0.533 ± 0.023	0.784 ± 0.010	0.664 ± 0.026
	GRAY	0.730 ± 0.010	0.520 ± 0.011	0.776 ± 0.006	0.655 ± 0.011
	RED	0.164 ± 0.002	0.311 ± 0.010	0.577 ± 0.001	0.483 ± 0.005
	GREEN	0.791 ± 0.007	0.603 ± 0.008	0.820 ± 0.003	0.705 ± 0.008
	BLUE	0.153 ± 0.004	0.347 ± 0.022	0.576 ± 0.003	0.476 ± 0.006
STARE	RGB	0.579 ± 0.077	0.348 ± 0.030	0.696 ± 0.020	0.497 ± 0.023
	GRAY	0.379 ± 0.146	0.312 ± 0.067	0.624 ± 0.041	0.487 ± 0.032
	RED	0.157 ± 0.004	0.444 ± 0.055	0.558 ± 0.010	0.456 ± 0.017
	GREEN	0.592 ± 0.085	0.442 ± 0.021	0.742 ± 0.010	0.517 ± 0.033
	BLUE	0.164 ± 0.005	0.327 ± 0.056	0.546 ± 0.013	0.474 ± 0.003
UoADR	RGB	0.323 ± 0.003	0.411 ± 0.004	0.699 ± 0.002	0.555 ± 0.004
	GRAY	0.319 ± 0.003	0.372 ± 0.019	0.679 ± 0.009	0.556 ± 0.005
	RED	0.238 ± 0.017	0.220 ± 0.014	0.598 ± 0.008	0.522 ± 0.005
	GREEN	0.328 ± 0.009	0.438 ± 0.019	0.713 ± 0.008	0.563 ± 0.004
	BLUE	0.262 ± 0.012	0.261 ± 0.008	0.619 ± 0.004	0.535 ± 0.002
